# A Novel Splice Site Mutation in *IFNGR2* in Patients With Primary Immunodeficiency Exhibiting Susceptibility to Mycobacterial Diseases

**DOI:** 10.3389/fimmu.2019.01964

**Published:** 2019-08-21

**Authors:** Aravind K. Bandari, Babylakshmi Muthusamy, Sunil Bhat, Periyasamy Govindaraj, Pavithra Rajagopalan, Aparna Dalvi, Siddharth Shankar, Remya Raja, Kavita S. Reddy, Manisha Madkaikar, Akhilesh Pandey

**Affiliations:** ^1^Institute of Bioinformatics, International Technology Park, Bangalore, India; ^2^Manipal Academy of Higher Education, Manipal, India; ^3^Center for Molecular Medicine, National Institute of Mental Health and Neurosciences, Bangalore, India; ^4^Pediatric Haematology, Oncology and Blood & Bone Marrow Transplantation, Mazumdar-Shaw Cancer Center, Narayana Health City, Bangalore, India; ^5^Neuromuscular Laboratory, Department of Neuropathology, National Institute of Mental Health and Neurosciences, Bangalore, India; ^6^National Institute of Immunohaematology, KEM Hospital Campus, Mumbai, India; ^7^Department of Laboratory Medicine and Pathology, Mayo Clinic, Rochester, MN, United States; ^8^Center for Individualized Medicine, Mayo Clinic, Rochester, MN, United States

**Keywords:** *IFNGR2* deficiency, gene therapy, IFN gamma signaling, non-tuberculous mycobacteria, infection, immunodeficiency

## Abstract

Primary immunodeficiency (PID) refers to a group of heterogeneous genetic disorders with a weakened immune system. Mendelian susceptibility to mycobacterial disease (MSMD) is a subset of PID in which patients exhibit defects in intrinsic and innate immunity. It is a rare congenital disorder characterized by severe and recurrent infections caused by weakly virulent mycobacteria or other environmental mycobacteria. Any delay in definitive diagnosis poses a major concern due to the confounding nature of infections and immune deficiencies. Here, we report the clinical, immunological, and genetic characteristics of two siblings (infants) with recurrent infections. There was a history of death of two other siblings in the family after BCG vaccination. Whole exome sequencing of the two affected surviving infants along with their consanguineous parents identified a novel, homozygous single nucleotide splice acceptor site variant in intron 2 of the interferon gamma receptor 2 (*IFNGR2*) gene. Sanger sequencing of DNA obtained from blood and fibroblasts confirmed the variant. The patients underwent bone marrow transplantation from their father as a donor. RT-PCR and Sanger sequencing of the cDNA of patients from blood samples after transplantation showed the expression of both wild type and mutant transcript expression of *IFNGR2*. To assess partial or complete expression of *IFNGR2* mutant transcripts, fibroblasts were cultured from skin biopsies. RT-PCR and Sanger sequencing of cDNA obtained from patient fibroblasts revealed complete expression of mutant allele and acquisition of a cryptic splice acceptor site in exon 3 that resulted in deletion of 9 nucleotides in exon 3. This led to an in-frame deletion of three amino acids p.(Thr70-Ser72) located in a fibronectin type III (FN3) domain in the extracellular region of IFNGR2. This illustrates individualized medicine enabled by next generation sequencing as identification of this mutation helped in the clinical diagnosis of MSMD in the infants as well as in choosing the most appropriate therapeutic option.

## Introduction

Mendelian susceptibility to mycobacterial disease (MSMD) is a rare and genetically heterogeneous immunodeficiency syndrome characterized by predisposition to severe and recurrent infections caused by vaccine against *Mycobacterium tuberculosis* (Bacillus Calmette-Guerin–BCG), which contains weakly virulent non-tuberculous mycobacteria and environmental mycobacteria (1). Predisposition to tuberculosis caused by *Mycobacterium tuberculosis* has also been reported in acquired or inherited immunodeficiencies (2). In addition, patients are susceptible to infections caused by other intracellular bacteria such as listeria and nocardia (3) and fungi such as candida (4), and histoplasma. Finally, viral infections caused by cytomegalovirus, human herpes virus 8 (5), parainfluenza virus type 3, respiratory syncytial virus (6), and varicella zoster virus (7) have also been reported in MSMD.

International Union of Immunological Societies (IUIS) PID expert committee has categorized MSMD as a defect in innate and intrinsic immunity (8). Owing to vaccination practices in many parts of the world, many newborns will receive BCG and those who have MSMD might be recognized as a consequence of this vaccination. The affected individuals have a predisposition to infections which manifests early in childhood and rarely in adulthood. These infections have been reported to affect soft tissues, bone marrow, lungs, skin, bones, and lymph nodes that may or may not recur (8).

The first case report of MSMD was published in 1951 (9) and the first report on its genetic etiology was published in 1996 with autosomal recessive inheritance (10). MSMD exhibits autosomal recessive, autosomal dominant, and X-linked recessive modes of inheritance (1). Mutations in 15 genes are currently known to cause MSMD, which include *IL12RB1, IL12B, IFNGR1, IFNGR2, STAT1, CYBB, IRF8, TYK2, ISG15, RORC, IKBKG, SPPL2A, JAK1, IL12RB2*, and *IL23R* (1, 11, 12). Most of these genes were reported with autosomal recessive mode of inheritance whereas *STAT1* was reported with autosomal dominant mode of inheritance. *IFNGR1* and *IRF8* have been reported with both autosomal dominant and autosomal recessive modes of inheritance. Of these genes, *IKBKG*, and *CYBB* are located on the X-chromosome lead to X-linked recessive mode of inheritance (13) (Table 1). Gene defects in *IL12RB1* have been reported most frequently followed by *IFNGR1* and *IFNGR2* (1). Approximately 40% of MSMD cases are due to mutations in *IL12RB1* and *IFNGR1* (14). The allelic heterogeneity of MSMD results in partial or complete defects in IFN-γ secretion, production, binding, or signaling (13).

**Table 1:** A list of the currently known 15 genes reported to be associated with Mendelian susceptibility to mycobacterial diseases.

**Gene symbol**	**Protein**	**Mode of inheritance**	**Defect**
*IL12B*	Interleukin 12B	AR	Complete deficiency with no mutant protein expression
*IL12RB1*	Interleukin 12 receptor, beta 1	AR	Complete deficiency with mutant protein expression
		AR	Complete deficiency with no mutant protein expression
*IKBKG*	Inhibitor of kappa light polypeptide gene enhancer in B-cells, kinase gamma	XR	Partial deficiency with mutant protein expression
*IFNGR1*	Interferon gamma receptor 1	AR	Complete deficiency and protein expressed
		AR	Complete deficiency and no protein expression
		AD	Partial deficiency and increased protein expression
		AR	Partial deficiency and protein expression
*IFNGR2*	Interferon gamma receptor 2	AR	Complete deficiency with protein level expression
		AR	Complete deficiency with no protein level expression
		AR	Partial deficiency with protein level expression
		AD	Partial deficiency with protein level expression
*STAT1*	Signal transducer and activator of transcription 1	AD	Partial deficiency; protein expressed but not phosphorylated
		AD	Partial deficiency; Mutant protein expressed but not bind to DNA
		AD	Partial deficiency; Mutant protein expressed but not phosphorylated or bind to DNA
*IRF8*	Interferon regulatory factor 8	AD	Partial deficiency with mutant protein expression
*CYBB*	Cytochrome b-245, beta polypeptide	XR	Complete deficiency with mutant protein expression
*ISG15*	ISG15 ubiquitin-like modifier	AR	Complete deficiency with no mutant protein expression
*RORC*	RAR-related orphan receptor gamma	AR	Complete IFNγ deficiency
*JAK1*	Janus kinase	AR	Complete and partial impaired response to IFNγ
*IL12RB2*	Interleukin 12 Receptor Subunit Beta 2	AR	Complete IFNγ deficiency with normal/ decreased protein expression
*IL23R*	Interleukin 23 receptor	AR	Complete IFNγ deficiency with normal/ decreased protein expression
*SPPL2A*	Signal Peptide Peptidase Like 2A	AR	Complete IFNγ deficiency with no/decreased/normal protein expression

*AR, Autosomal recessive; AD, Autosomal dominant; XR, X-linked recessive*.

*The genetic etiology of these genes such as autosomal recessive (AR), autosomal dominant (AD) or X-linked recessive (XR) and the functional consequences of the mutation whether it is a complete or partial loss of the protein along with its expression are described*.

Early detection of infection and accurate diagnosis of MSMD is important for better clinical outcomes. Careful and thorough physical examination and history, especially about consanguinity, and similar complaints in the family are important clues to diagnosing primary immunodeficiency (15). Presence of certain microbial infections throughout the body can further aid diagnosis (15). Laboratory investigations such as blood counts and antibody titers are essential to rule out other causes of immunodeficiency (15). The overall prognosis for MSMD is poor. Patients with MSMD do not generally respond to external IFN-γ as they lack functional receptors and hence they survive on antibiotics alone (16). Hematopoietic stem cell transplantation (HSCT) is the only treatment for patients with absent IFN-γ (14, 17, 18). Studies have revealed that the transplants from HLA matched individuals carry a high risk of graft rejection owing to the presence of high IFN-γ concentrations in the plasma of the patients (19, 20).

In this study, we report the clinical, immunological and genetic manifestations of two infants with MSMD born of consanguineous parents. We carried out whole exome sequencing of the proband, his affected brother along with both asymptomatic parents, and identified a novel splice site mutation in *IFNGR2* gene as a potentially genetic cause for MSMD observed in the family. RT-PCR and Sanger sequencing of the cDNA obtained from skin fibroblasts confirmed loss of the conventional splice acceptor site and acquisition of an alternate cryptic splice acceptor site in the exonic region which resulted in an in-frame loss of 9 nucleotides.

## Materials and Methods

### Ethical Statement

This study was carried out in accordance with the recommendations of “ICH-GCP, Indian Council of Medical Research guidelines & Revised Schedule Y Guidelines of Indian Drugs and Cosmetics Rules 1945,” Narayana Health Medical Ethics Committee with written informed consent from all subjects. All subjects gave written informed consent in accordance with the Declaration of Helsinki. The protocol was approved by the Narayana Health Medical Ethics Committee.

### Exome Sequencing and Data Analysis

Whole exome sequencing and data analysis were carried out as described previously (21). In addition, PROVEAN was used to predict the effect of protein altering indels (22). Further, the shortlisted variants were subjected to segregation analysis using autosomal recessive mode of inheritance which was regarded as the most likely pattern of inheritance based on disease segregation in the family. The shortlisted variants were manually reviewed for potential association of identified mutations in immune related functions. Sanger sequencing of genomic DNA was carried out in a region spanning the splice acceptor site mutation identified in *IFNGR2* gene in patients and their parents. The following are the primers used for sequencing: forward: 5′-CCTCAGCACCCGAAGATTC-3′; reverse: 5′-TTGAAACCAAGGCATTGTCAC -3′.

### Transcript Analysis of Blood Samples After Bone Marrow Transplantation

Blood samples were collected in PAXgene blood RNA tubes and total RNA was extracted using the PAXgene blood RNA kit using standard protocols (catalog number # 762164). We used about 1 μg RNA to synthesize single stranded cDNA using High Capacity cDNA Reverse Transcription Kit (Catalog number # 4368814) as per the manufacturer's instructions. In order to validate and study the consequences of the splice site mutation identified in *IFNGR2* gene, we designed primers (F: 5′-GCTCCTCAGCACCCGAAGAT-3′; R: 5′-AGCGATGTCAAAGGGAGAGGA-3′) spanning a region of 405 bp in *IFNGR2* transcript (RefSeq: NM_005534.3). This region is amplified using RT-PCR and the products were run on an agarose gel to visualize the expression of the amplicon. Further, PCR products were cleaned up using QIAquick PCR Purification Kit and the products were quantified and Sanger sequencing was carried out.

### Fibroblast Culture and RNA Extraction

Skin biopsy was performed using a 3 mm needle from both the affected siblings. Fibroblasts were grown from the skin biopsies *in vitro* at 37°C in a 5% CO_2_ atmosphere in Dulbecco's modified eagle medium (DMEM) with sodium pyruvate and L-glutamine (Gibco #12800-017) and supplemented with sodium bicarbonate, 20% fetal bovine serum, and 1% antibiotic antimycotic solution. Cells were expanded until full confluency and harvested. Total RNA was extracted from the fibroblasts using an RNeasy mini kit. High capacity cDNA reverse transcriptase kit was used for cDNA synthesis for RT-PCR analysis. The PCR products were visualized by agarose gel electrophoresis. Sanger sequencing was performed using the primers described above.

## Results and Discussion

**Figure 1:**
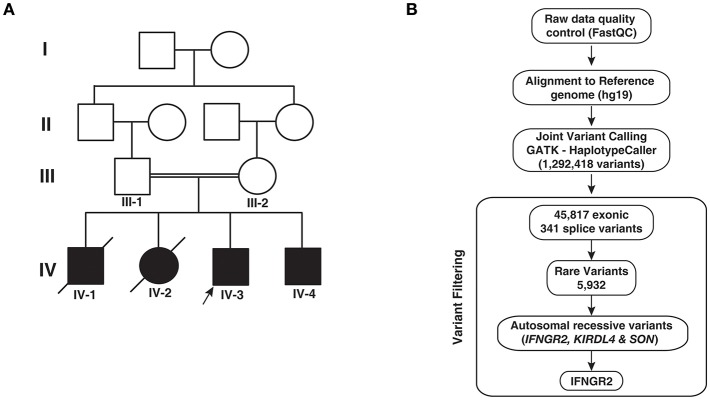
Pedigree and analytical workflow. **(A)** Pedigree showing affected and unaffected individuals across four generations. Exome sequencing was carried out in the proband (IV-3) and his sibling (IV-4) and both parents (III-1 and III-2). **(B)** Depiction of the workflow adopted to identify potential causative variant from whole exome sequencing data.

In this study, we recruited an Indian family with two affected siblings born of consanguineous parents. Two other elder siblings in the family died after BCG vaccination. The proband (IV-3) was a third born infant of consanguineous parents (Figure 1A). At six months of age, the patient showed symptoms of cervical swellings, abdominal distension, and failure to gain weight. At nine months, he developed fever followed by a persistent cough. BCG vaccination was not administered to this patient because of the adverse effect of BCG observed in the older siblings. The CD4:CD8 ratio was found to be normal with increased CD45 counts. He was normal for a month and after that multiple swellings in the neck, axilla, and groin were observed. The symptoms continued to progress and the child lost weight in spite of a good appetite. There was gradual distension of the abdomen accompanied by dry and scaly skin changes. He was passing stools 4–5 times a day which were watery, non-bulky, non-greasy, and semi-solid. He had severe pallor, dependent edema, and features suggestive of congestive cardiac failure (CCF). Following blood transfusions and management of CCF the patient improved. Child also received nutritional support and multivitamins. Patient had a reactive marrow and therefore, not suggestive of lymphoma, or dimorphic anemia with neutrophilic leucocytosis. Chest X-ray was normal, ultrasound studies of the abdomen showed hepatosplenomegaly with mesenteric lymphadenopathy. Hemoglobin electrophoresis was normal. TORCH, a screening test for toxoplasmosis, others (HIV, hepatitis viruses, varicella, parvovirus), rubella (German measles), cytomegalovirus, and herpes simplex was normal. Lymph node biopsy showed reactive lymphadenitis with some histiocytes. Both TB-PCR and Mantoux tests were negative. Other investigations revealed decreased platelets and increased C-reactive protein (CRP) levels. Evaluation for HLH was normal, EBV-PCR was negative, and lymph node culture for AFB (BACTEC) revealed no growth. Haplotype-matched bone marrow transplant from the father was performed due to severe, recurring infections in the patient. The patient is stable and is cured of his disease.

The younger sibling (IV-4) of the proband (IV-3) is the fourth affected child in the family (Figure 1A). As in the case of IV-3, IV-4 manifested symptoms at 9 months. He had fever and enlarged lymph nodes in the neck. BCG vaccine was not administered owing to the adverse reactions observed in the elder deceased siblings. A haplotype-matched bone marrow transplant from the father was performed and he is also cured of his disease.

The first born (IV-1) was a male infant delivered after a normal pregnancy. He had an adverse reaction to BCG upon vaccine administration. The first infection was an episode of cold and cough followed by left axillary and cervical swelling at 3 months. Because of the prevailing fever, he was hospitalized for 20 days. Abdominal distension and hepatosplenomegaly were observed. The infant passed away at 6 months of age. The second born (IV-2) was a female infant who also had an adverse reaction to BCG vaccine. The infant developed swelling in the left axilla and was diagnosed to have disseminated BCG lymphadenitis. She was suspected to have T-cell deficiency and the lymph nodes were removed; however, the open wound ulcerated and the she died at 4 months of age.

### Whole Exome Sequencing and Analysis

In order to identify potential genetic cause for the phenotypes observed in patients, we performed whole exome sequencing of the two surviving infants and their parents. We obtained an average of 64 million reads of which ~99% were aligned to the human reference genome (hg19). We obtained an average depth of 78× across the four individuals. After post alignment quality measures, we carried out joint variant calling across the four members. We obtained a total of 1,292,418 variants. Gene annotation of these variants using Annovar resulted in the identification of 45,817 exonic and 341 splice site variants, which were considered for further downstream analysis. Common variants with minor allele frequency >0.01 were discarded after comparing the variants with 1,000 genomes project, ExAC, and EVS resulting in 5,932 variants. Filtering variants based on autosomal recessive pattern of inheritance resulted in three variants that corresponded to a missense variant in *SON*, a frameshift causing insertion in *KIR2DL4* (rs11371265), and a novel splice acceptor site variant in *IFNGR2* (Table 2; Figure 1B). SON is a DNA binding protein involved in splicing and its role in immune related functions is not known.

**Table 2:** Mutations identified in this study that are inherited as autosomal recessive mutations.

**Gene symbol**	**Locus**	**Genomic change**	**Amino acid change**	**Type of mutation**	**Effect on protein function**
*IFNGR2*	Chr21	g. 34793786G>A	p.(Thr70-Ser72)	Splice site	Deleterious
*KIR2DL4*	Chr19	g. 55324674C>CA	p.S267fs	Frameshift causing insertion	Probably benign
*SON*	Chr21	g.34925531C>G	p.Pro1332Ala	missense	Probably benign

A single nucleotide insertion c.802dupA resulting in a frame shift in *KIR2DL4* gene (RefSeq: NM_001080772.2) was identified in homozygous state in both patients and heterozygous state in the consanguineous parents. *KIR2DL4* gene encodes for killer cell immunoglobulin like receptor, two Ig domains and a long cytoplasmic tail 4, located at 19q13.42. It belongs to a family of killer cell immunoglobulin-like receptors (KIRs), which bind to HLA class I molecules and are involved in innate immunity. *KIR2DL4* is a membrane bound receptor of natural killer cells and reported with 53 alleles in IPD-KIR database (release: 2.7.1), of which two common alleles known as 10A and 9A have been reported with controversial expression and function (23). The allele 10A encodes the full protein with 10 adenine nucleotides located in the part of the gene that encodes the transmembrane domain and the 9A allele generated by a single adenine nucleotide deletion causes a frameshift which results in premature termination of the protein product (23). These genotypes influence cell surface expression of *KIR2DL4* (23). Instead, we identified an 11A allele with a homozygous insertion of an adenine after the 10th adenine, which corresponds to a transmembrane domain in KIR2DL4. In contrast to the 9A allele, the 11A allele led to the elongation of the protein product. The resulting protein was 377 amino acids long that is similar the protein encoded by a known isoform of *KIR2DL4* (RefSeq: NM_002255.6). Thus, this variant is not a deleterious variant, which could explain the immunodeficiency observed in the patients.

### *IFNGR2* Splice Site Variant

**Figure 2:**
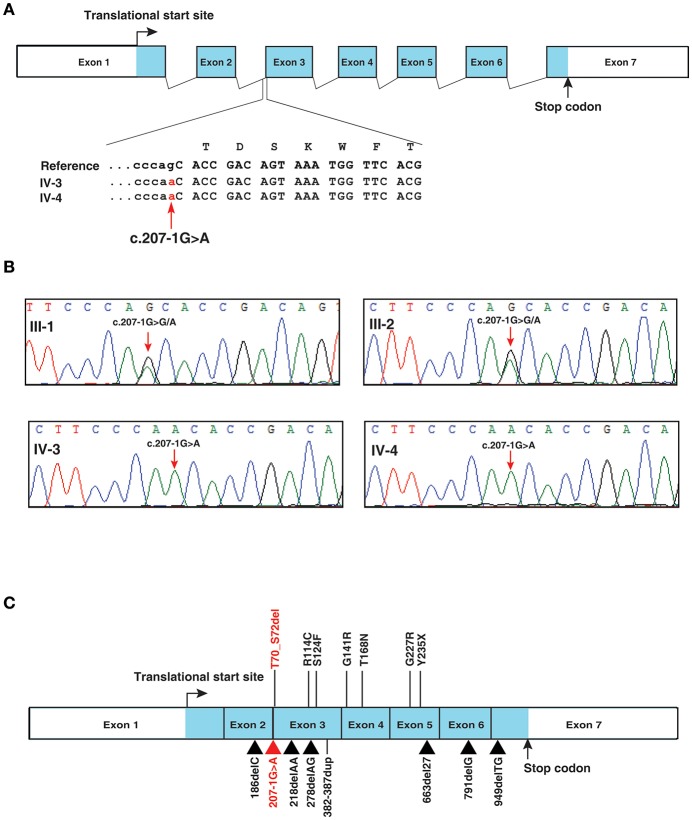
*IFNGR2* splice site mutation. **(A)** Depiction of *IFNGR2* splice acceptor site mutation in intron 2 of *IFNGR2* gene. The changes shown are homozygous in the affected siblings **(B)** Illustration of segregation of the splice site mutation identified in *IFNGR2* gene (this study) in the affected siblings and unaffected consanguneous parents using Sanger sequencing. **(C)** Depiction of currently known mutations and the novel splice site mutation (this study) in the exon-intron architecture of *IFNGR2* gene. The splice acceptor site mutation identified in this study is marked in red and the previously reported mutations in *IFNGR2* gene are marked in black.

A novel splice site variant c.207-1G>A (chr21:g.34793786G>A) was identified in intron 2 of *IFNGR2* gene (RefSeq: NM_005534.3) (Figure 2A). *IFNGR2* gene encodes for interferon gamma receptor 2 located at 21q22.11. This variant was not reported in dbSNP, 1,000 genomes project or ExAC. MutationTaster predicted the effect of this variant as disease causing (Prediction: “D”; Score: 1). In addition, the official OMIM entry (OMIM: 147569) for the *IFNGR2* gene confirms its association with Immunodeficiency 28 (IMD28) (MIM #614889), a primary immunodeficiency disease causing a genetic predisposition to mycobacterial infections by atypical Mycobacteria and the BCG vaccine (1). Of note, family history of adverse reaction to BCG vaccination was documented for two deceased older siblings of the affected individuals. This is the first study to document a possible disease-associated mutation in the splice site of *IFNGR2*. Sanger sequencing of a region around the *IFNGR2* splice acceptor site variant (c.207-1G>A) confirmed the presence of the mutation in both affected individuals as homozygous and in the parents as heterozygous (Figure 2B). Thus, these mutations may underlie autosomal recessive MSMD with partial or complete deficiency. Previous studies implicating *IFNGR2* in MSMD have described mutations in exonic regions affecting intracellular, transmembrane, and extracellular domains of IFNGR2 (Figure 2C) (24–32).

### Transcript Analysis to Study the Consequences of the Splice Acceptor Site Mutation

**Figure 3:**
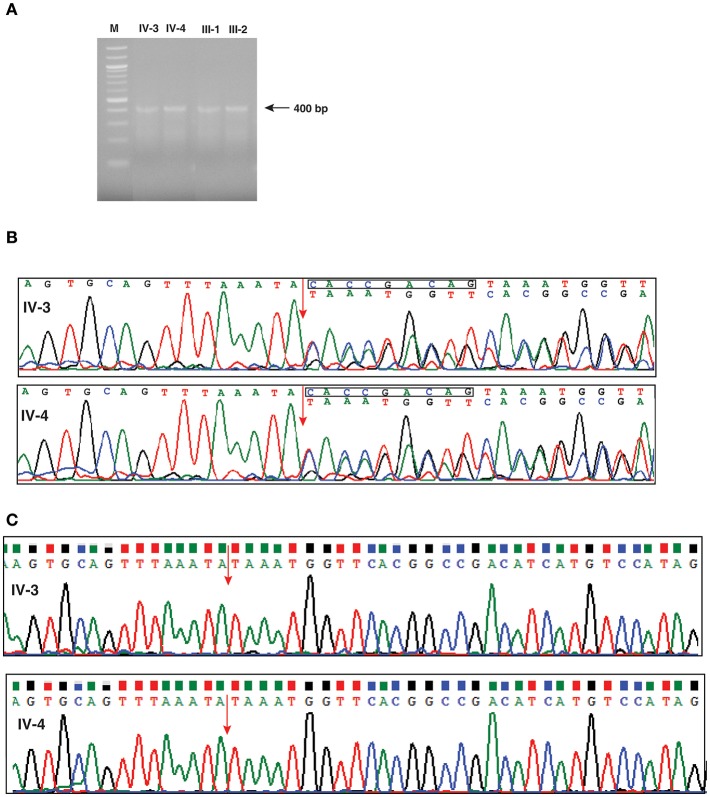
Investigation of effect of the splice defect using reverse transcription PCR and Sanger sequencing. **(A)** Agarose gel image of the RT-PCR products targeted for a region spanning the splice acceptor site mutation of *IFNGR2* in patients and unaffected parents. The gel image shows a single band around 400 bp consistently across the patients and unaffected parents. **(B)** Depiction of the effect of splice site mutation using chromatograms obtained from the Sanger sequencing. The splice junction between exon 2 and exon 3 is shown by an red arrow. The chromatogram shows double peaks after splice junction depicting the existence of both normal and mutant *IFNGR2* transcript. The nucleotide sequences of the normal (top) and mutant transcripts (bottom) are shown. The deleted nucleotides in mutant transcripts are shown in a rectangle in the normal transcript. **(C)** Sanger sequencing results of *IFNGR2* gene isolated from patients skin fibroblast cells. The red arrow indicates the splice site of exon 2 and 3 and the sequencing data reveals deletion of nine nucleotides. Two panels represents the sequencing data of patients IV-3 and IV-4.

In order to investigate any potential molecular defect caused by the identified splice acceptor site mutation from whole exome analysis, we again collected blood samples from the patients, and parents. However, the blood samples collected this time were subsequent to bone marrow transplantation. We isolated total RNA from the samples and carried out RT-PCR analysis. The RT-PCR product was run on an agarose gel and we observed that all four samples consistently showed a single band of ~400 bp, which corresponded to the expected target of 405 bp (Figure 3A). We carried out Sanger sequencing of the PCR products for all four samples. Because of the splice site mutation, either exon skipping could occur or an alternate cryptic splice acceptor could be created either in intronic or exonic region of the gene. Analysis of the Sanger sequencing results consistently showed two peaks per nucleotide after the splice junction in both affected parents and patients showing the expression of both the mutant and normal forms of *IFNGR2* gene owing to the fact that the blood samples were collected after bone marrow transplantation for this analysis (Figure 3B). Subtracting the peaks that correspond to the normal transcript, we reconstructed the mutant *IFNGR2* transcript sequence based on the alternate peaks observed in the Sanger sequencing data (Figure 3B). Further, we used BLAT tool and visualized the results in a browser to verify the location of the reconstructed sequence of the alternate transcript and found that it was located after 9 nucleotides in the exon 3 downstream of the splice acceptor site mutation. Thus, the normal splice acceptor site is destroyed and an alternate cryptic splice acceptor site was created within exon 3.

Since the transcript analysis of *IFNGR2* gene in patients' blood samples showed expression of both wild type and mutant alleles (Figure 3B), we wanted to check the expression of wild type *IFNGR2* expression in patients fibroblast cells where no mixing with donor cells could occur. For this, we obtained skin biopsy samples from both the patients and cultured fibroblasts using standard procedures. We first isolated DNA from these cultured fibroblast cells of both the patients and amplified them using the same primers used for blood DNA amplification and carried out Sanger sequencing. We reconfirmed the homozygous splice site mutation in both the patients. In order to check the transcript level expression of *IFNGR2*, we also isolated RNA from the cultured fibroblasts and generated cDNA for amplification using the same set of primers used for transcript analysis of blood samples. Sanger sequencing showed expression of only the mutant allele confirming the 9 nucleotides deletion (Figure 3C).

This novel splice acceptor site mutation causes deletion of three amino acids p.(Thr70-Ser72) in *IFNGR2* (RefSeq: NP_005525.2). This deletion occurs in the FN3 domain and in Tissue_fac domain which are in the extracellular region of the receptor. The region spanning amino acids 70–73 belongs to one of the variable loop regions in the protein structure that is assumed to control the binding specificity of *IFNGR2* (33). PROVEAN predicted the effect of this deletion mutation as deleterious (Score: −5.935).

### Role of *IFNGR2* in MSMD

*IFNGR2* encodes for the interferon gamma receptor 2 located at 21q22.11. Human interferon-gamma receptor is a heterodimer of *IFNGR1* and *IFNGR2* (34). Both *IFNGR1* and *IFNGR2* are synthesized in endoplasmic reticulum and are transported to the golgi apparatus (35). *IFNGR2* gene is composed of 7 exons which span ~33 kb of genomic DNA. Exon 1 and exon 2 encode a signal peptide while exons 2, 3, 4, and 5 encode for the extracellular domain. Exon 6 encodes a part of extracellular domain and the transmembrane domain while exon 7 encodes the intracellular domain and 3′UTR.

More than 27 patients have been reported thus far with several different etiologies described including (i) autosomal recessive or dominant inheritance, (ii) abolished or maintained expression of *IFNGR2*, (iii) complete or partial *IFNGR2* deficiency with or without cell surface expression, (iv) expression of non-functional *IFNGR2* on the cell surface, (v) creation of new glycosylation site that causes misfolding of the protein that abolishes the cellular responses, among many other etiologies (36).

In the first ever reported case of *IFNGR2* deficiency resulting in MSMD (24), the case history documented persistent cough and subsequent lymphadenopathy, hepatosplenomegaly, and fevers. A similar clinical presentation was observed in the proband in this study. Progressive weight loss and lymph node biopsy positive for histiocytosis were additional similarities observed between this case and confirmed MSMD cases with either complete or partial *IFNGR2* deficiency resulting from mutations in extracellular domain of the *IFNGR2* gene (24, 25) (Figure 2C).

Of note, while no appreciable clinical response to subcutaneous IFNγ treatment was observed in the case of complete *IFNGR2* deficiency, symptoms owing to partial *IFNGR2* deficiency could be significantly alleviated with IFNγ administration (27, 29). It is interesting to note that there are two reports of MSMD resulting from complete *IFNGR2* deficiency-causing mutations that could still be rescued with IFNγ administration (27, 28). In both cases, the mutations resulted in surface expression of non-functional *IFNGR2* with abnormal N-glycosylation sites. The clinical consequences in patients with complete deficiency of *IFNGR2* are often severe or fatal.

The homozygous form of *IFNGR2* splice acceptor site defect that we have observed is likely to be pathogenic leading to a potential loss of function of *IFNGR2* as suggested by the death of two siblings in the family. Given that the transplant was successful and the patients are doing well at the age of seven and 5 years respectively, it seems that a combination of thorough clinical history taking, appropriate diagnosis and the use of next generation sequencing for confirming the molecular aberration can help in choosing the most appropriate therapeutic option in affected individuals.

## Conclusions

Our study underscores the importance of employing NGS-based platforms for a global exploration of primary immunodeficiencies to identify gene mutations in a clinically relevant time frame. Such studies will also aid in a more thorough understanding of the genetics of immune disorders and possibly lead to additional screening in newborns in the future. The splice acceptor site mutation identified in this study that leads to deletion of three amino acids require additional functional studies to understand the functional implications of this mutation in immune related functions.

## Data Availability

The raw data supporting the conclusions of this manuscript will be made available by the authors, without undue reservation, to any qualified researcher.

## Ethics Statement

This study was carried out in accordance with the recommendations of ICH-GCP, Indian Council of Medical Research guidelines Revised Schedule Y Guidelines of Indian Drugs and Cosmetics Rules 1945, Narayana Health Medical Ethics Committee with written informed consent from all subjects. All subjects gave written informed consent in accordance with the Declaration of Helsinki. The protocol was approved by the Narayana Health Medical Ethics Committee.

## Author Contributions

AP, SB, and MM conceptualized and designed the study. BM and PR wrote the initial draft of the manuscript. AB wrote the sections of manuscript, sample collection, processing, and performed the experiments. BM analyzed the data. SB and MM diagnosed the patients and performed the immunological assays. AD assisted in the immunological assays. PG cultured the fibroblast cells. RR helped AB for performing experiments. KR participated in the initial design of the project. All authors contributed to manuscript revision, read and approved the submitted version.

### Conflict of Interest Statement

The authors declare that the research was conducted in the absence of any commercial or financial relationships that could be construed as a potential conflict of interest.
